# Successful Response of Pembrolizumab Rechallenge after Radiotherapy for a Patient with Bladder Cancer of Nonresponse of Pembrolizumab First Challenge

**DOI:** 10.1155/2021/9087529

**Published:** 2021-07-28

**Authors:** Taro Ikeda, Go Hasegawa, Gen Kawaguchi, Yohei Ikeda, Noboru Hara, Tsutomu Nishiyama

**Affiliations:** ^1^Department of Urology, Uonuma Institute of Community Medicine, Niigata University Medical and Dental Hospital, Minamiuonuma, Niigata, Japan; ^2^Department of Pathology, Uonuma Institute of Community Medicine, Niigata University Medical and Dental Hospital, Minamiuonuma, Niigata, Japan; ^3^Department of Radiotherapy, Uonuma Institute of Community Medicine, Niigata University Medical and Dental Hospital, Minamiuonuma, Niigata, Japan; ^4^Department of Diagnostic Radiology, Uonuma Institute of Community Medicine, Niigata University Medical and Dental Hospital, Minamiuonuma, Niigata, Japan

## Abstract

We report a patient with advanced bladder cancer in which the primary lesion and metastatic site disappeared following the pembrolizumab therapy rechallenge after radiotherapy for bladder cancer lesion of nonresponse of pembrolizumab first challenge. A 76-year-old man with advanced bladder cancer received three courses of the chemotherapy with gemcitabine and cisplatin combination; however, the chemotherapy was stopped because of adverse events. The patient started pembrolizumab therapy; however, the effect was not observed. Radiation therapy was given to the primary lesion and pelvic lymph node metastases for the purpose of local control of the lesions. Because the primary lesion was regrowth and para-aortic lymph node metastasis appeared, pembrolizumab therapy was resumed. Thereafter, the primary lesion and metastatic site disappeared.

## 1. Introduction

Although the treatments of advanced bladder cancer have made significant progress in recent years, most patients go on to have progressive disease and are still incurable [1]. We report a patient with advanced bladder cancer in which the primary lesion and metastatic site disappeared following the pembrolizumab therapy rechallenge after radiotherapy for bladder cancer lesion of nonresponse of pembrolizumab first challenge.

## 2. Case Report

**Figure 1:**
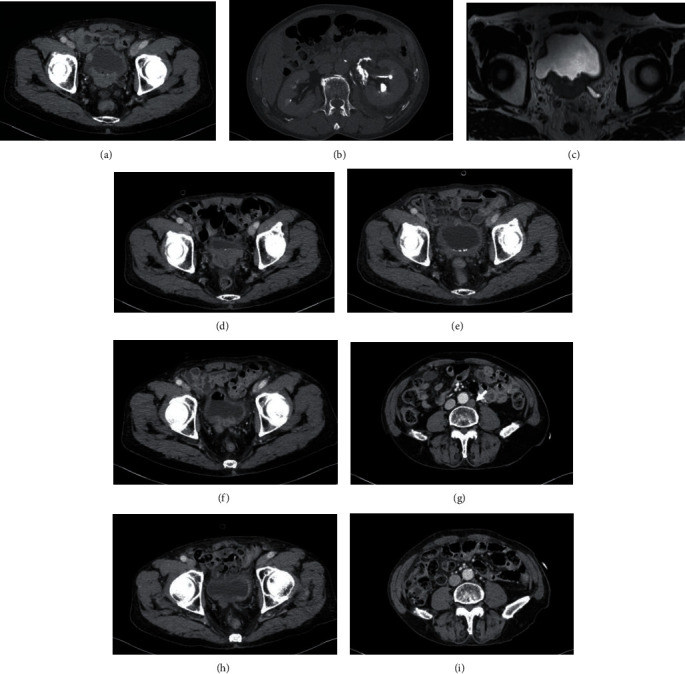
Imaging findings. (a) CT in April 2019 at diagnosis shows the extramural invasive tumor in posterior and trigonal bladder wall. (b) CT urography at diagnosis shows the leakage of contrast media because of extravasated urine. (c) MRI at diagnosis shows that the extramural invasive tumor in posterior and trigonal bladder wall is a low signal intensity on T2-weighted image. (d) CT in December 2019 at the discontinuation of pembrolizumab therapy shows growth in the primary lesion. (e) CT in June 2020 after radiotherapy shows diminished primary tumor in size. (f) CT in September 2020 three months following radiotherapy shows regrowth of the primary lesion. (g) CT in September 2020 three months following radiotherapy shows the emergency of para-aortic lymph node metastasis (arrow: para-aortic lymph node metastasis). (h) CT in February 2021 at the end of 8 doses of pembrolizumab therapy resumption shows significant reduction of the primary lesion. (i) CT in February 2021 at the end of 8 doses of pembrolizumab therapy resumption shows almost disappearance of para-aortic lymph node metastasis.

**Figure 2:**
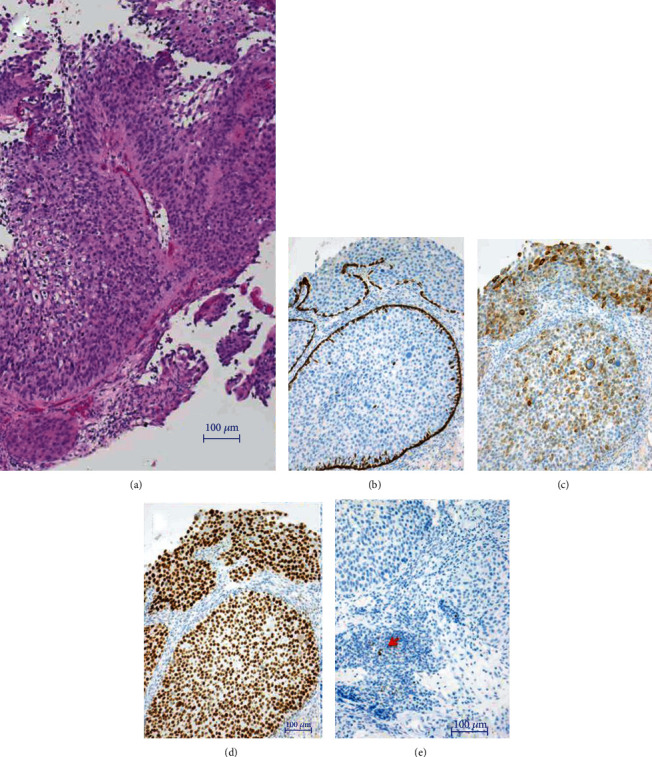
Pathological findings. Pathological findings revealed invasive urothelial carcinoma (UC) (luminal type UC, papillary, G2, high grade) (H&E stain (a)), CK5/6 negative (b), CK20 weak positive (c), GATA3 strong positive (d), and PD-L1 negative (red arrow: macrophage) (e).

A 76-year-old man was pointed out microhematuria and left hydronephrosis during treatment for rheumatoid arthritis at a nearby doctor and was referred to our hospital in April 2019. Cystoscopy showed a solid mass from trigonum to the posterior wall of the bladder, and bilateral ureteral orifices could not be confirmed. Contrast-enhanced computed tomography (CT) of the thoraco-abdomino-pelvis revealed bladder cancer with extramural invasion and left hydronephrosis and left renal urine extravasation due to left ureteral obstruction of exponential growth of bladder tumor (Figures 1(a) and 1(b)). Bladder magnetic resonance imaging (MRI) revealed bladder cancer with extramural invasion (Figure 1(c)). Bladder tumor biopsy revealed invasive urothelial cancer and high grade (Figure 2). With the diagnosis of left hydronephrosis and left renal urine extravasation due to left ureteral obstruction at the left ureteral orifice with invasive bladder cancer, left nephrostomy was performed urgently. The patient underwent gemcitabine/cisplatin combination therapy (GC therapy) (70% dose in consideration for his age). The effect of three courses of GC therapy was stable disease. Continuing GC therapy was planned but was stopped because low-grade fever continued, and CT showed shadows in both lung fields. The patient started pembrolizumab therapy (pembrolizumab 200 mg intravenously, every 3 weeks) in September 2019 after confirming improvement in general condition and remission of shadows in both lung fields. The patient was noted to have a gastric ulcer after four courses of pembrolizumab therapy and discontinued pembrolizumab therapy due to regrowth in the primary lesion and the appearance of pelvic lymph node metastases in December 2019 (Figure 1(d)). The patient was found to have increased primary lesion and pelvic lymph node metastasis on CT in March 2020. The patient underwent radiotherapy (50.4 Gy with 28 fractions) on the primary lesion and pelvic lymph node metastasis site for the purpose of local control of the lesions. The primary lesion and pelvic lymph node metastasis site diminished in size after the radiotherapy (Figure 1(e)). The patient subsequently resumed pembrolizumab therapy due to regrowth of the primary bladder lesion and the appearance of para-aortic lymph node metastasis on CT in September 2020 (Figures 1(f) and 1(g)). At the end of eight doses of pembrolizumab therapy in February 2021, the primary lesion and para-aortic lymph node metastasis were significantly reduced and had maintained (Figures 1(h) and 1(i)). In May 2021, the patient was placed a ureteral stent in the left ureter, and the left nephrostomy catheter was removed. Immune-related adverse event due to pembrolizumab therapy has not been observed.

## 3. Discussion

We experienced a patient with advanced bladder cancer without any effect of the first pembrolizumab therapy in which the primary lesion and metastatic site disappeared by rechallenge of pembrolizumab therapy after radiation therapy was applied to the primary lesion. We could not find out a urothelial cancer case report of successful response of immune checkpoint inhibitor (ICI) rechallenge after radiotherapy, following discontinuation of ICI because of its ineffectiveness. Until now, almost all patients reported with ICI rechallenge after discontinuation of ICI were the patients experienced discontinuation of ICI due to the emergence of immune-related adverse events [2].

Platinum-based chemotherapy is the preferred initial approach for systemic therapy in patients with metastatic bladder cancer and most patients with muscle-invasive bladder cancer who will undergo a radical cystectomy in neoadjuvant chemotherapy setting [1, 3]. In the present patient, GC therapy was performed for three courses but was stopped because of adverse events.

Advances in bladder cancer treatment have been unprecedented thanks to ICI immunotherapy [4]. ICI immunotherapies continue to displace previous treatment regimens as first-line and second-line therapies in bladder cancer [4, 5]. ICI immunotherapies unfortunately are not effective in all patients with advanced bladder cancer, and many patients go on to have progressive disease. Various attempts have been made to improve the therapeutic effect of ICIs. One of the modalities is the combined use of radiation therapy [6]. Radiation therapy has been shown to enhance the priming and effector phases of the antitumor T-cell response rendering it an attractive therapy to combine with PD-1/PD-L1 inhibitors; however, the sequencing, dose, fractionation, and targets for radiation therapy are still investigations. The many ongoing clinical trials on the subject will help answer many practical questions related to radiation therapy. The abscopal effect has drawn attention for the phenomenon by which systemic antitumor responses are observed outside of the primary site of local irradiation [7]. It has been described in a number of different malignancies, including metastatic renal cell carcinoma, melanoma, and hepatocellular carcinoma. In the present patient, the primary lesion regrowth and new lesions appeared after irradiation, and the tumor shrank due to retreatment with pembrolizumab, which is unlikely to be the abscopal effect. Wu et al. demonstrated that the staining of PD-L1 was significantly linked with lower completed response rates, higher loco-regional failure rate, and reduced disease-free survival and lower survival with intact bladder for patients treated with definite completed chemoradiotherapy [8]. Radiation therapy may have been responsive well to the tumor because the tumor tissue was PD-L1 negative in the present patient.

There are mechanisms of action that have the potential to turn cold tumors into so-called hot and inflamed tumors, hence increasing the tumor's responsiveness to immunotherapy-increasing local inflammation, neutralising immunosuppression at the tumor site, modifying the tumor vasculature, targeting the tumor cells themselves, or increasing the frequency of tumor-specific T cells [9]. Radiation therapy for the purpose of local tumor disease control may have led to increasing inflammation in the tumor microenvironment. The present patient resumed pembrolizumab therapy due to regrowth of the primary bladder lesion and the appearance of para-aortic lymph node metastasis and by reference to an accumulating body of evidence showing the immunomodulatory role of radiation therapy and its increased efficacy when combined with immunotherapy. It is considered that the rechallenge of pembrolizumab therapy worked effectively for shrinkage of the regrowth of bladder tumor and the para-aortic lymph node metastasis after radiotherapy in the primary lesion. In the future, it is hoped that the mechanism of action of combination therapies centered on ICIs will be elucidated, and reliable methods for enhancing the effectiveness of ICIs will be established.

## 4. Conclusion

The patients may be able to expect the positive effect on regression of the tumor for pembrolizumab rechallenge if they respond to radiation therapy to the tumor. Reliable methods for increasing the effectiveness of ICIs will expect to be established.
